# Making public ahead of print: Meetings and publications at the Royal Society, 1752–1892

**DOI:** 10.1098/rsnr.2016.0030

**Published:** 2016-09-07

**Authors:** Aileen Fyfe, Noah Moxham

**Affiliations:** School of History, University of St Andrews, St Andrews, Fife, KY16 9BA

**Keywords:** Science, publication, communication, orality, sociability, pre-print

## Abstract

This essay examines the interplay between the meetings and publications of learned scientific societies during the eighteenth and nineteenth centuries, when journals were an established but not yet dominant form of scholarly communication. The ‘making public’ of research at meetings, long before actual ‘publication’ in society periodicals, enabled a complex of more or less formal sites of communication and discussion ahead of print. Using two case studies from the Royal Society of London—Jan Ingen-Housz in 1782 and John Tyndall in 1857 to 1858—we reveal how different individuals navigated and exploited the power structures, social activities and seasonal rhythms of learned societies, all necessary precursors to gaining admission to the editorial processes of society journals, and trace the shifting significance of meetings in the increasingly competitive and diverse realm of Victorian scientific publishing. We conclude by reflecting on the implications of these historical perspectives for current discussions of the ‘ends’ of the scientific journal.

## Introduction: What counted as publication?

In 1782, Jan Ingen-Housz, in Vienna, sent a hastily written paper to the President of the Royal Society of London, Joseph Banks, ‘trust[ing] upon your friendly sentiments for me, that you will get it re[a]d’ at a meeting.^1^ Seventy-five years later, James David Forbes, in Edinburgh, described himself as waiting in ‘inuendo and suspense’ to learn what had really been argued at a recent meeting of the Society.^2^ In both cases, men of science from distant cities were avidly interested in the meetings of the Royal Society: Ingen-Housz wanted his research read at the meeting, and Forbes wished to know in detail what had happened. Their interest is a clear indication of the important role of the meetings of the Society to eighteenth- and nineteenth-century science.

We suggest that this is an opportune moment to reconsider the functions of learned society meetings. Scholars of scientific societies, correspondence networks, salons and *conversazioni* have shown how, at various times, these were all valuable means of communicating natural knowledge among scholars and other audiences.^3^ We are interested, here, in the interplay between meetings and publication; and, particularly, in the role of society meetings during a period in which journals were an established but not yet dominant form of academic communication.^4^

At the Royal Society, and organizations like it, research was ‘made public’ long before actually being ‘published’ in print in the Society's transactions or memoirs. Mario Biagioli has examined this space between ‘making public’ and ‘publication’ from the perspective of priority and ownership, leading him to refer to it as a ‘phase of heightened vulnerability’ for authors.^5^ But the implications are broader-ranging than this suggests. First, the meetings opened up a range of possibilities for the public and quasi-public discussion, dissemination and refinement of natural knowledge long before the printed *Transactions* appeared. Second, any analysis of the selection and editorial processes of scientific knowledge needs to start with those who were involved in organizing the meetings, not editors and referees. Processes of discussion and comment began prior to publication, inside and outside the Society, running parallel to and sometimes feeding into the process of publication. Third, the relationship between meetings and publications was not static: over time learned societies changed their procedures, and the book trade (and thus the context and competition) changed around them.

In this article, we focus on the Royal Society between 1752 and 1892. Printed journals were part of a complex ecosystem of ways of making research public, and enabling subsequent discussion, conversation, comment and revision. 1752 marks the Royal Society's assumption of management of the *Philosophical Transactions*, and the official requirement that all research communicated to the Society be presented first at a meeting, and only later considered for publication in the *Transactions*.^6^ Despite increasing internal pressure to reform and external pressure from new and competing modes of scientific print, Society meetings retained their primacy throughout this period. In 1892, however, a new set of standing orders formally acknowledged that, due to the press of papers submitted and the limited time available in meetings, papers could be considered for publication even if all that was read of them in the meetings was the title.^7^

Our aim is not simply to point out that learned society meetings have been significant means of communicating scientific knowledge. Rather, we investigate how such meetings functioned, and how they related to print publication, in an age when the variety of forms of print publication was increasing, and certain forms of print were becoming essential to scholarly reputation-building. We will start by explaining how the relationship between the Royal Society's meetings and publications changed over the eighteenth and nineteenth centuries. We next discuss two case studies: Jan Ingen-Housz's experiences in the 1780s illustrate how meetings were organized and papers shaped by the power of the Society's officers, and demonstrate the importance of personal influence and connections; while James David Forbes's frustration in the 1850s, as he waited for details of John Tyndall's paper on glaciers, enables us to illustrate the range of public and quasi-public discussion that arose after the meeting and before publication.^8^ We will then consider the challenges created by this mixed ecosystem of meetings and publications, and the ongoing usefulness of Royal Society meetings despite growing competition from new forms of print. We conclude by reflecting on the implications of these historical perspectives for current discussions of the ‘ends’ of the scientific journal.

## From primacy of meetings to primacy of print at the Royal Society

The weekly London meeting had been the irreducible element of the Society's activity since its foundation in 1660. As conceived by the founding Charters and Statutes, meetings were intended as sites of natural-philosophical experiment and discussion.^9^ This twin emphasis, and the experiments shown at meetings in the 1660s and 1670s by Robert Hooke, Christopher Wren, Robert Boyle and Denis Papin, among others, are well known.^10^ The gleanings of natural-philosophical news, letters, books and discoveries from beyond London, which made up much of the early *Philosophical Transactions*, represented a secondary activity in the eyes of the Society, as one of us has argued elsewhere.^11^

By the early 1750s the situation had changed. Experiment had disappeared from meetings altogether, and discussion had waned. Meetings were given over instead to the reading of ‘papers’—research communiqués authored by Fellows and non-Fellows of the Society. A selection of these would be published by the editor of *Transactions* (by custom the Society's Secretary, but operating outside its oversight). The tenuous seventeenth-century link between what was published in *Transactions* and what went on in Society meetings became much stronger during the first half of the eighteenth century, and the official takeover of the periodical by the Society in 1752 made the relationship absolute.^12^ Editorial control was transferred from a succession of single individuals to a Committee of Papers: a reorganization that was intended to shield the Society, collectively and individually, from the imputation of negligence, nepotism or malpractice, but which also had the effect of marking each paper published with an institutional stamp of approval. From 1832, one or more Fellows was also asked to report in writing on papers being considered for publication in *Transactions*; but the Committee of Papers remained the ultimate decision-making body. *Transactions* usually appeared in biannual parts, so that most individual papers appeared in print many months after they had first been communicated.

In this arrangement, every paper read to the Society was to be considered for publication; equally, no paper was to be considered for publication without being read to the Society. Only Fellows could communicate research to a meeting, though they could do so on behalf of others. This was the most obvious form of gatekeeping, which clearly favoured those with personal connections to the Fellowship: friendship and family connection were established routes for getting the attention of the President or Secretary, and thus bringing a paper to the attention of the Society. Even after the sheer volume of incoming papers enforced changes to the standing orders in the 1890s, the rule about communicators remained in force.

The Society's claim to print the materials communicated to its meetings was an expression of ownership, and is discernible at many levels, from the Society's record-keeping practices to its public relations. Tracing its ramifications reveals both the complexities of particular cases and broad changes in the commercial, social, professional and organizational contexts of British science as well as the Society's own practices and priorities. There were, for example, several instances in the late eighteenth and early nineteenth centuries when the Society was dismayed to learn of papers approved for publication in *Transactions* finding their way into print in other venues, including London newspapers and the new commercial scientific journals.^13^ The greater uptake and circulation of ‘separate copies’—later known as offprints—by authors had increased the danger of a *Transactions* paper being anticipated in print. By the nineteenth century, their circulation through an author's correspondence networks had become a thoroughly established aspect of the ways in which new scientific research spread; but the Society printed a warning on the cover asking recipients to do their best to ‘prevent them from being reprinted till one Month after the publication’ of *Transactions* itself.^14^ This was all the more necessary because of a fundamental change in the landscape of scientific print: the multiplication of increasingly reputable sites for the publication of scientific work. As well as commercial monthly magazines devoted to the sciences, there were the publications of the new learned societies and a variety of daily, weekly and monthly periodicals that sometimes reported scientific news.^15^ Some of these sites offered alternative routes to publication that did not entail the built-in delay between communication and publication associated with *Transactions*; and others provided routes for some of the content of Society meetings to circulate before publication of *Transactions*.

One way to obviate the threat of being scooped in this way was for the Society to create its own, more rapidly appearing source of news. From 1831 it began publishing abstracts of all papers read at recent meetings, effectively redoubling the link between meetings and papers.^16^ Known as *Proceedings*, this periodical marks the Society's acceptance that there was a need for information about what went on in the Society to circulate before (and beyond) *Transactions*. The Society's publication of meeting reports helped drive another important change in the meetings–papers relationship. As we will see, the selection and ordering of material for meetings was not subject to collective control or Society oversight, but was left to the judgement of the President and Secretaries. This important power was widely understood and potentially manipulable, and Society officers were accused of abusing it at different times throughout the period in view. Once it became the case that having a paper accepted for reading at a meeting virtually guaranteed publication, even if only in brief form in *Proceedings*, there was correspondingly more at stake in the initial screening process, from both the author's and the institution's point of view. New standing orders of 1892 publicly articulated for the first time the criteria that would be used for choosing papers for meetings (papers which ‘the author is prepared to illustrate by experiments, diagrams &c., or which is likely to give rise to discussion’); but in doing so, the Society, also for the first time, relaxed the condition that tied publication to meetings.^17^ Henceforth a subset of papers accepted for publication would be read at a meeting, rather than a subset of the papers read at the meeting being published. It was, we suggest, a critical symbolic moment, representing a tacit acknowledgement of the subordination of meetings and the primacy of print publication.

## Gatekeeping, presidential influence and the organization of meetings under Joseph Banks

Royal Society meetings in the 1780s were regimented affairs—in contrast to the noisy, slovenly chaos described by John Hill in one of his series of satires on the Society from the early 1750s. Hill's writings are frequently supposed to have helped spur the Society to assume formal editorial control of *Transactions*.^18^ The orderly conduct of meetings in the latter part of the eighteenth century is often ascribed to the personal style of Joseph Banks (President from 1778 to 1820).^19^ One of Banks's early initiatives as President was to move Society meetings to later in the evening—to 6 p.m. from 8 p.m.—and to limit them to an hour's duration.^20^ Among other things, this permitted members of the Royal Society Club, and their guests, to dine together ahead of the meeting.^21^

The slightly pressurized running of Society meetings under Banks was therefore partly a function of the limited time available in the meetings themselves, coupled with the fact that the Society only met between the beginning of November and the end of June (so that a paper communicated towards the end of June might have to wait until the following November to be read aloud). The officers had to make choices about which papers to prioritize, how much time to spend on any given communication, and how much of it to read out. Some papers were split between meetings, and others read in abstract rather than in full.^22^ This power of sequencing, cutting and shaping papers for the Society vested considerable additional influence in the Society's officers, beyond simple gatekeeping. This de facto concentration of power over meetings and publications was one of the chief bones of contention in the early dissatisfaction with Banks's leadership of the Society. (The other was his opposition to discussion within meetings.)^23^

Experienced authors were well aware of the inbuilt rhythms of Society activity, and of the eccentricities of its underlying power structures. One notable instance from the early 1780s is the case of Jan Ingen-Housz (1730–1799), a Dutch-born chemist and physician, who had lived in London in the 1770s and had been a Fellow of the Society since 1769. His important discoveries about plant respiration—and specifically the crucial point that the sun's light and not its heat was an indispensable factor in plants’ production of dephlogisticated air (i.e. oxygen)—were published in *Experiments upon Vegetables*, printed at London in 1779.^24^ In the early summer of 1781, Joseph Priestley, the English chemist, dissenting minister and a former experimental collaborator of Ingen-Housz's, published a second volume of his own *Experiments and Observations* in which he claimed this central discovery for himself, and dedicated a short section of the book to a disparagement of the apparatus on which Ingen-Housz's results depended—Felice Fontana's eudiometer—and Ingen-Housz's use of it.^25^ The incident was made worse by the London *Critical Review*'s enthusiastic reception of Priestley's work and its patronizing dismissal of Ingen-Housz.^26^

By the spring of 1782, Ingen-Housz—now in Vienna—had heard of Priestley's claims and was complaining of them to his correspondents. He dashed off a paper in defence of his discoveries and his methods, and sent it to Banks on 6 May, asking him to ‘get it re[a]d before the recess at the R.S.’. Ingen-Housz's awareness of the Society's seasonal cycle made him particularly anxious about the timing, not least because the post took over a fortnight to reach London. He was planning a second edition of *Experiments upon Vegetables*, and feared it would be vitiated in advance in the eye of the scientific public if Priestley's ‘flat overturn of my doctrine’ were allowed to go unchallenged for too long. He wanted it to ‘be known among the philosophers that I can defend my cause’, but needed this to happen swiftly. Ingen-Housz told Banks that ‘if it should not be re[a]d before the breaking up of the society’, he would consider ‘whether it be not advisable to publish it by itself’, but feared that such delay ‘would be a finishing Strook [sic] for a whole year’ to his publication plans.^27^ France and Germany both had commercial journals specializing in chemistry and physics, but there were no credible alternative English sites for publication: the first British commercial scientific periodicals were still 15 years away. Ingen-Housz clearly hoped that his paper would be read before the Society and printed in *Transactions*. The appearance, at least, that his self-vindication should have been heard and approved for publication in an official forum—the principal assembly of men of science in England—was crucial to his strategy against Priestley.

Banks's reply was friendly and frank, but non-committal. Explaining his usual rationale for deciding the running-order of meetings, he expressed himself willing to have the paper read before the recess, but doubted ‘whether it will be in my power to do it consistent with the general Justice Expected from me in arranging the papers for reading’. Banks tried to balance ‘the importance of the paper & the priority of delivery’: the order in which papers had been received against the importance of the research they contained. Two of the remaining three meetings were already fully committed, as well as part of the final meeting. Banks estimated that Ingen-Housz's paper ‘will take 35 minutes reading at least’, and that was slightly more than the time available. He could not in fairness give Ingen-Housz ‘a preference over all subjects deliverd in long ago’, unless some ‘accident’ with another author gave him ‘an opportunity to push it forward’.^28^

Whether other papers were withdrawn, or whether Banks thought Ingen-Housz's results were especially important, or whether he thought Priestley had been unjust, is impossible to say—but Ingen-Housz's paper was indeed read to the Society before the recess, and duly published in *Transactions*. Matters were certainly helped by Banks's finding a reason to make some spontaneous cuts to the paper, writing to Blagden on 16 June that Ingen-Housz had sent ‘some good Experiments’ mixed with ‘no small abuse upon priestly’. Banks reported that he had ‘cut out the Abuse & read the Experiments to the Soc who will probably order them to be printed’.^29^ Blagden replied to the effect that he thought Banks had done well to keep the Society out of the controversy, while observing speculatively that Ingen-Housz's strictures upon Priestley may not have been unwarranted: ‘So many things of the same kind have fallen upon this latter Gentleman, that one is led to suspect he goes as near the boundary line as possible, if he does not sometimes tread over it.’^30^

This episode gets into small compass many of the issues surrounding the conduct of meetings and their importance as a site for making research public. First, there is Ingen-Housz's familiarity with the logistical constraints on Society meetings and their link to publication (and above all to the speed of publication), and his understanding of the prestige the Society could confer by hearing and printing a paper. Second, there is his understanding that if he wished to see his paper pushed speedily up the Society's agenda it was sensible to address the request to the President, with whom he was on good if not necessarily intimate terms. Third, it makes plain Banks's wish to navigate controversial subjects diplomatically but even-handedly where possible, and hints at the awareness of the Society's officers of certain scholars’ reputations for being difficult customers. It also affords an instance of unilateral editing prior to reading at a meeting on the part of the Society's officers—which is reflected in the paper as printed^31^—and it is evidence for the need to plan meetings and sequence papers carefully and with an eye to time.

Ingen-Housz's distinctive problem lay in needing to establish a defence of his research before a credible audience—specifically an English one, since it was in England, and with English audiences, that Priestley's critique was most likely to resonate—and in having to do so from a distance that prevented his participation in the social world of London science. Meetings of the Society were embedded in an informal but well-established framework of scientific sociability, including Thursday morning ‘philosophical breakfasts’ at Joseph Banks's house in Soho Square (even in Banks's absence) and the dinners of the Royal Society Club. Such events afforded plentiful opportunities for networking, building alliances and discussing research, before and after it was communicated to an official meeting. Blagden and Banks evidently used the breakfasts to plan the Society's business, including the running of meetings; and Blagden's diary shows that new papers were circulated and discussed at the dinners.^32^ Cut off from these by distance, Ingen-Housz was at a disadvantage. Nonetheless, his earlier involvement with the Society meant that he had the personal influence and tacit knowledge needed to ensure that his response to Priestley through a meeting of the Society would be understood to have been validated, if not necessarily formally endorsed, by the London natural-philosophical world.

## Glacial manoeuvrings: John Tyndall, J. D. Forbes and making knowledge public in the 1850s

Many of the features of Royal Society meetings in the late eighteenth century remained current in the mid nineteenth. Dining and socializing continued to be associated with the Society: presidential breakfasts still happened, though less frequently, perhaps because of the gulf in social rank between the aristocratic Presidents and the bulk of the active membership; soirees were part of the Society's calendar; and a second, more exclusive, dining club was founded in 1847 ‘to facilitate intercourse’ among active researchers.^33^ The Society still met every Thursday in the season, and meetings still lasted about an hour. The members of the new Royal Society Philosophical Club met for dinner at 5.30 p.m., and from 7 p.m. engaged in discussion about the upcoming paper and any other ‘scientific subject worthy of consideration’.^34^ Since part of the rationale was to encourage attendance and participation at the meetings, they then adjourned to the Society meeting at 8 p.m.

The long-standing constraints on time were exacerbated by the growing number of papers submitted to the Society, and a new desire to encourage discussion and to show experiments. The introduction of discussion occurred during the reforms of the 1840s, and was particularly necessary in light of competition from the newer, discipline-based societies.^35^ Thus, in early 1853, just months before he was elected to the Fellowship, the physicist John Tyndall reported that after the reading of his paper on the transmission of heat:

A gentleman arose and made a kind of critical Speech, closing it by a certain question which he said he should like to ask the author … I got up and replied. It was the first time I had opened my mouth in the Society and I was totally unprepared, but nevertheless what I said seemed to please them for when I sat down they gave me a round of applause.^36^

On that occasion, Tyndall claimed, he had ‘chanced to drop in to the Royal Society’ without ‘the slightest anticipation’ that his paper was to be read.^37^ Later in the 1850s, he would have been able to check the published schedule of papers in the *Literary Gazette*.^38^ By then, Tyndall had been appointed a professor at the Royal Institution, where experimental demonstrations were an important aspect of his public lectures. Where these involved new research, he often presented them to the Royal Society audience first; but Tyndall was well aware of the time constraints, telling the President on one occasion: ‘Ten minutes would suffice to read my communication if it be not accompanied by experiments. But half an hour at least would be needed if experiments be introduced.’^39^

Although the arrangement of the meetings continued in broadly similar fashion, the wider communications context of Victorian Britain was very different from that of the 1780s, when the *Philosophical Transactions* had been almost the only scientific periodical in Britain.^40^ By the 1850s it was one among many and the Royal Society was one scientific society among many. Royal Society meetings and publications no longer had any realistic pretensions to dominate the communication of natural knowledge. The new landscape of scientific print meant that discoveries could be put into print more quickly in many other periodicals; and it also meant that discoveries communicated to the Society could be more widely discussed in print as well as in manuscript ahead of their formal print publication in *Transactions*. Here, we investigate how this worked for a paper presented to a Royal Society meeting in 1857.^41^

In Britain, the study of glaciers was associated with the Edinburgh University professor James David Forbes.^42^ In 1857, a paper on the structure and motion of glaciers was communicated to the Royal Society by John Tyndall and his friend Thomas Henry Huxley, though no one doubted it was Tyndall's work.^43^ Tyndall intended it as an attack on Forbes and his theory, telling a friend a few weeks earlier that ‘I think I have smashed up this theory’ and ‘utterly annihilated it’.^44^ The disagreement between Forbes and Tyndall continued into the 1860s, and has previously been examined both for its role in the study of the long history of the earth, and as part of the tensions between London-based Tyndall and the North British men of science.^45^

The official date for the ‘making public’ of Tyndall's theory was 15 January 1857. There are two notable things about this, both pointing to the ongoing significance of personal connections and sociability. First, at least some of the London-based Fellows had already heard something of Tyndall's investigations two months earlier, because he spoke about them at a dinner of the Royal Society Philosophical Club on 13 November.^46^ Presumably the limited audience and the brief, informal nature of the talk meant that this was not regarded as pre-empting the communication of the paper to a meeting of the Society (as a full paper delivered to a learned society, or a Royal Institution lecture would have done).

Second, the printed version of the paper that (eventually) appeared in *Transactions* claims that the paper was both received and read on 15 January.^47^ Usually, a paper was scheduled for a meeting only once it had been submitted: one of the other papers read on 15 January had been received on 27 November, and other papers received in mid January were not read until late February or March.^48^ Thus, the official printed dates suggest that Tyndall used his personal influence to secure a slot before he had finished writing the paper. Moreover, he may not have submitted the written version until even later, given that the ‘Register of Papers’ in the Society's archive gives 29 January as the date received.^49^ Again, this points to the special position held by those with close links to the inner circle of the Royal Society. (Although relatively junior, Tyndall had recently become a member of Council.)

We know little about what happened at the meeting itself. Tyndall reported to his friend the next day that the ‘hour and a half talking at the Royal Society last night’ had made him ‘very weary’.^50^ Charles Darwin was one of those present and claimed to have been ‘most deeply interested … I can hardly get [the] subject out of my head’. But he had been ‘forced to retreat from my head aching so’, and thus wrote to Huxley two days later to assuage his curiosity about something that ‘I daresay came into the tail of the paper’.^51^

An eight-page ‘abstract’ of the paper was printed in the Society's *Proceedings* in mid February; but it was not until late May that Tyndall learned it would be printed in full in *Transactions.*^52^ It was too late to appear in Part I of the 1857 volume, so although, as we shall see, Tyndall was able to circulate printed copies of the paper among his correspondents in October, formal publication did not happen until Part II of the 1857 volume appeared, sometime between November 1857 and February 1858.^53^ However, Tyndall's correspondence makes clear that a great deal of discussion and reaction to the paper had taken place long before Part II of *Transactions* appeared. Word spread via a mixture of conversation, lectures given by Tyndall, correspondence and newspaper and magazine reports. These media varied in the details they contained, and in the publics who had access to them. Thus, different people knew different things at different times.

One of those most interested in the paper was, of course, Forbes. He was a Fellow of the Society but as an Edinburgh resident (and long-time Secretary of the Royal Society of Edinburgh) he more frequently attended meetings of the Royal Society of Edinburgh than of London. Nevertheless, he had his contacts in London. He first learned about Tyndall and Huxley's paper from a letter written by Edward Sabine (Treasurer of the Society, 1850–1861) to an Edinburgh correspondent the day after the meeting. Forbes told one of his correspondents later that month:

My first knowledge of the London world being stirred on the glacier theory came in not the most pleasant manner. Two letters arrived one after the other from a London celebrity, whom I shall not at present name, to my colleague, Balfour, evidently glorying in my coming defeat in terms sufficiently provoking, but which showed that it was to be made a regular party question in scientific circles there.^54^

Forbes now had a problem: he knew he was being attacked and talked about in London, but he knew very little of what was actually being said. If he was to craft a response, he needed to know the basis of the critique. As he wrote: ‘Regarding Mr. Tyndall and his theory, I have received no such precise information as to enable me to speak confidently about it’. He knew that he would, in a month or so, receive *Proceedings of the Royal Society*; but in the meantime, he ‘wrote to Sir Charles Lyell for some information about it, which he kindly gave me’. Even so, Forbes still lacked the full picture. While Darwin, who was on friendly terms with Huxley, felt able to write directly for further information, Forbes did not: ‘All I can do is to sit still till the indictment is made out, and I cordially wish my enemy to write a book and print it speedily, as anything is better than inuendo and suspense.’^55^

While Forbes waited impatiently for *Proceedings*, those in London were learning more. Tyndall gave a related lecture, ‘Observations on Glaciers’, at the Royal Institution on 23 January, which was reported at some length in the *Saturday Review* on 31 January and in the *Literary Gazette* on 7 February.^56^ In contrast, the Royal Society's meetings were rarely reported in detail, suggesting that Joseph Banks's opposition to reprinting ahead of the full *Transactions* publication was still largely respected. The *Philosophical Magazine* did not report the January meetings of the Society until September (and even then did not mention Tyndall's paper).^57^

Tyndall circulated the press reports of his Royal Institution lecture among his correspondents, sending copies to Rudolf Clausius in Zürich,^58^ Carlo Matteucci in Pisa,^59^ Augustus de la Rive in Geneva^60^ and John Herschel in Kent. Tyndall's covering letter to the latter, in early March, acknowledged that Herschel would probably have ‘already seen the substance of it in the Proceedings’, but Tyndall used the opportunity to try to impress the eminent natural philosopher.^61^ It is clear that Tyndall was an effective self-promoter; but also that circulating journalistic reports of his public lecture at the Royal Institution was not seen as an infringement of the Royal Society's right to be the first to publish the full paper.

If Forbes saw the reports in the *Saturday Review* or the *Literary Gazette*, he did little with them. He probably saw the paragraph in his local *Chambers's Journal* on 28 February, but it would have added little to what he had heard from Lyell.^62^ He was waiting for the somewhat fuller account that would be in *Proceedings*,^63^ and thus it was not until 6 March that he felt able to contact Tyndall, writing: ‘My dear Sir, I received lately the number of the Proceedings’. He wrote that he planned to make ‘some remarks’ on it, but would prefer to know more detail. Aware of the processes of the Royal Society, he asked if Tyndall yet knew ‘whether it is to be printed’, and if so, ‘will you oblige me by sending me a copy of it as early as convenient?’.^64^ Tyndall wrote back the next day promising a copy at ‘the earliest opportunity’ if it were indeed printed. He added that he trusted Forbes would find ‘the fullest recognition’ of his own earlier research, and ‘no expression at variance with the Philosophic Spirit’.^65^ Forbes then spent time in March experimenting on ice, and reported his findings to the Royal Society of Edinburgh.^66^ But it was not until October that Tyndall was finally able to send Forbes the full paper, for, as he said, ‘the Royal Society is very slow in printing its memoirs’.^67^

The debates about the physical properties of ice and the motion of glaciers rumbled on through 1857 and 1858, with more magazine articles and lectures from both sides. In terms of ‘making public’, however, two things are notable about this episode: the commercial periodical press was very slow to report Royal Society meetings;^68^ and as far as one can tell from Tyndall's correspondence, the eventual publication in *Transactions* did not herald a new rush of interest. It appears that most of the interested parties had heard about it long before ‘official’ publication in winter 1857–1858.

## Getting ahead: the perils and benefits of meetings in an age of publications

These two episodes reveal how the Royal Society's meetings were organized and controlled, and how they functioned to make public new claims to natural knowledge. There are many similarities between the two cases in terms of organization and gatekeeping, but there are significant differences in attitudes to time and speed. We now reflect on the problems and advantages of communicating research to a meeting of the Society, given both the tight link to print publication and also the period of delay until that publication.

The function of Society meetings, as a necessary first step for publication, means that the visible editorial processes do not tell the whole story of how the Society's approbation was granted.^69^ In one sense, the rules were clear: research could only be communicated by Fellows, or by those vouched for by a Fellow. But the actual mechanisms for deciding who appeared on the schedule, on which day and in what level of detail, remained informal throughout the period we have examined. Thus, an extremely important aspect of the running of the Society was left in the hands of the President and Secretaries, and largely beyond the scrutiny of the rest of the Fellowship. This invisible gatekeeping depended on the officers’ sense of the importance of research, of the justice required of a particular situation, even of a paper's social propriety and scientific civility. Edward Sabine's confession in 1869 that, as President, he generally recused himself from the selection and ordering of papers for meetings made little difference; as we have seen, Tyndall was able to exploit his reputation and connections to secure the slots he wanted and the length of time he wanted for speaking, in a manner that suggested little change since the age of Banks.^70^ Inasmuch as a pattern can be discerned, this situation particularly benefited a small number of high-profile, productive, well-connected men of science—not necessarily all in London. Where it generated complaints, these tended to come from particular spheres.^71^

The biggest concerns about the relationship of meetings and publication were surely about timing, and, particularly in the later period, about the gap. Jan Ingen-Housz was certainly worried about fitting in before the summer recess, yet it was the date of the meeting that was important to him; he was planning over a time-frame of a year or so, at a time when there were fewer opportunities for public discussion between the meeting and the publication. For Victorian men of science, working with publication time-frames of months or weeks and a flood of new options for scholarly communication, the delay between meeting and print was clearly a potential source of acute anxiety and frustration.^72^ For Forbes, the delay made it difficult for him to defend himself against Tyndall's critique. (His wish that his ‘enemy would write a book and print it speedily’ implied an impatience with the plurality of forms of scientific communication, and almost suggests a nostalgia for the scientific book—for the singular, monolithic expression of his opponent's views, and a static target in controversy.)

Another category of problem arose from the possibility of changes being introduced to a paper between the meeting and publication. In the 1780s, it was common practice—and an unquestioned right—for the Society's Officers and the Committee of Papers to revise a submitted paper as they saw fit, either before or after communicating it to the meeting—witness Banks's unilateral excision of Ingen-Housz's abuse of Priestley. The general rule seems to have been that what was published in the Society's periodicals should not differ substantively from what the members heard in its meetings. If two different ideas of the same paper were to circulate in the public domain, one through the recollection of its hearers and the other through the understanding of its readers, there was a clear potential for confusion if not controversy. It could also vitiate the function of the date of communication as a means of registering discovery and recording priority.

The increased use of refereeing from the 1830s might also be seen to encourage the introduction of changes between meeting and publication. Referees often asked for cuts in the introduction and conclusion of papers, or requested clarification of particular terms. The Secretaries nevertheless insisted that such changes be ‘merely verbal’—in other words, not intellectually substantive. When, in 1848, it was feared that an author had made more significant changes, the Secretary felt it would be necessary to add a note giving the date of the changes, thus modifying the significance of the original ‘date read’.^73^ As refereeing became more established, authorial revisions were increasingly expected, but nonetheless, Tyndall was present at a Council meeting in 1880 which reiterated ‘the rule’ that ‘any substantial changes … should be properly dated’.^74^ On the face of it, this is an odd limitation to have imposed on referees and notably different from modern conceptions of the function of peer review. It becomes intelligible if we understand how rigorously the Society wished to enforce the identity between meeting and publication, expecting referees to recognize and operate within that framework.

Despite the problems that could arise from communicating research in a forum that made it quasi-public rapidly, but insisted on a substantial delay before full publication, there were certain advantages to making public at a meeting. For Ingen-Housz it was faster than negotiating with a printer to issue a pamphlet, let alone a book. (He might have written something for Lorenz Crell's *Chemische Annalen*, had it been the German-speaking world he wished to influence.) But by the 1820s, a short communication to the editor of the *Philosophical Magazine* could appear in print in less than a month; and after the establishment of *Nature* in 1869, it could appear the following week. The weekly meetings of learned societies were no longer a uniquely speedy way of making public. As Melinda Baldwin has noted, an abstract or letter in *Nature* was no substitute for a lengthy and well-illustrated paper in *Transactions*, but it was an excellent way of spreading the word about the general tenor of one's findings. Where Tyndall used his Royal Institution lectures and the literary weekly magazines to promote his theory ahead of full publication, the following generation would use *Nature*.^75^

For those who could be in London in person, a clear advantage of communicating to a meeting was social. Not only would one meet other Fellows at the meeting itself, but, as we have seen, the Society was the hub of a plethora of social activities. For younger men in particular these opportunities for seeking advice, intellectual input and patronage from senior Fellows were invaluable. Recognition of the value of social face-to-face interaction can similarly be seen in enthusiasm for the annual meetings of the British Association for the Advancement of Science, which brought together men of science from all over the country, much as academic conferences would later do.^76^

Besides, having one's work published by the Royal Society conferred intellectual prestige. A Victorian man of science who merely wanted a date-stamp and attribution for his discoveries could publish in a commercial science monthly, but the Royal Society structures offered something more. In particular, being chosen for publication in *Transactions* was a mark of approbation, not just by an editor, but by the Society as a whole, thanks to collective editorial processes quite unlike those at the commercial journals.^77^ By the mid nineteenth century, this reputational benefit associated with *Transactions* clearly outweighed the limited communicative power of the slow and stately printed volumes themselves.

The intriguing thing is that communicating to a Royal Society meeting also carried significant cachet. On one level, throughout this period, the inclusion of a paper in a Society meeting actually signified little more than that time was available in the schedule, and that its author was a Fellow or known to a Fellow (or, failing that, the paper seemed interesting to one of the officers). Nonetheless, Ingen-Housz thought that being heard with respect by Britain's most venerable scientific assembly was a public token of approbation; and Tyndall was clearly pleased with the applause he received at his first, unplanned, performance at the Society in 1853. In the eighteenth century, scholarly reputations were routinely made on the basis of oral conversation and social repute, and could thus be forged in meetings regardless of publication.^78^ By the late nineteenth century, scholarly reputations were made through publications, preferably in prestigious periodicals; and the fashion within learned societies was for procedures to be set down in writing rather than improvised by officers. Thus, while meetings continued to be run the old-fashioned way, the practices for newly important publications were becoming increasingly codified. Meetings retained their prestige for historic reasons, and as a necessary precursor to possible publication. After the 1890s, speaking at a meeting or a conference remained valuable for social reasons and for obtaining advance news of the latest research, but it was publication that built reputations.

## Public Knowledge: The End(s) of the Scientific Journal

The symposium at which this paper was originally delivered asked participants to envisage ‘the end of the scientific journal’. Implicit in this formulation was the question of whether the scientific journal remains fit for purpose, and what, in the modern world, those purposes should be. There are some clear parallels between our historical case studies and the contemporary situation: then as now, a flood of new options challenged the traditional modes of scholarly communication. The modern emphasis on the list of publications as a crucial marker of accomplishment for professional academic scientists has overshadowed what we have tried to illuminate here, namely the extent to which research was ‘made public’ at meetings ahead of print deep into the nineteenth century. The modern dominance of the scientific journal article relegated other forms of scholarly communication to the wrong side of a sharp divide, to be derogated as merely ‘informal’ or ‘in progress’. This distinction is now being intentionally eroded by new online tools such as pre-print servers, which do not merely improve the reach and speed of scientific communication, but also recreate opportunities for quasi-public discussion in the space between ‘making public’ and ‘publication’.

In an important sense, our analysis locates the original emergence of that divide. We have traced a transition from the primacy of face-to-face scientific meetings as the basic unit of research communication to the primacy of the printed article by the end of the nineteenth century: or, more precisely, to a tacit admission on the part of the Royal Society that its historically preferred principle for structuring the communication of scientific research was no longer tenable by 1892. By then, the scientific environment in Britain had fundamentally changed. Where *Philosophical Transactions* and the Society itself had virtually no English competitors when Ingen-Housz wrote to Banks, the numbers of scientific actors, in the form of new learned societies, new sites of scientific discussion, new universities and technical colleges, new scientists and new periodicals, commercial and otherwise, had vastly increased by the end of Tyndall's life. The growing importance of publication for individual reputation and advancement, and the increasing number of people wishing to publish, surpassed the capacity of weekly Society meetings to act as a filter for scientific publication. Those factors also produced a formalization of editorial practices—much more among learned society publications than their commercial competitors, ironically enough.^79^ The flexible mechanisms by which scientific work reached print in the age of Banks hardened into increasingly standardized protocols as the ideal of equitable and meritocratic access for authors to the publication process, and its rewards, spread.

It may be argued that the invention of the scientific journal, in its modern sense, must be dated to the point at which the scientific journal became not only the chief instrument by which scientific workers communicated with one another, but also at which publication in journals became indispensable to a scientific career. The year 1892 seems to us as useful a point in time as any to locate this, with the proviso that the Royal Society was responding belatedly to prevailing conditions rather than driving them.^80^

It is also important to note that making natural knowledge intelligible and accessible to the non-scientific public is *not* one of the traditional ends of the scientific journal, according to this definition. Yet this is at the centre of contemporary arguments for open access, and is one of two principal challenges to the traditional model of scientific communication—the other being that online forms of dissemination make the print-and-paper form and part-issue structure of the traditional journal largely unnecessary. We profess no special attachment to either the traditional form of the scientific journal or to the traditional definition, but would ask whether such an obligation to the public, which funds much scientific research, can be reconciled to the traditional communicative function of journals and to their role in professional accreditation. This is, after all, the question at stake in the debate about the future of scholarly communications. The pursuit of publication in high-prestige periodicals, condemned as invidious by many scholars and by advocates of open access in particular, nevertheless remains for the present a crucial component in the progression of academic careers. The relevance of historical perspectives to these questions is sharply apparent, because if those markers of prestige are removed, as open access threatens to do, the professional advantage of academic scientists is in effect sacrificed to the public's right to be informed of the research it funds. Without an alternative structure for bestowing scholarly approbation and professional reward, the entire basis for academia has to be reimagined. The logic of open access and the thoroughly disinterested pursuit and communication of natural knowledge at its heart hearkens back, consciously or not, to before the nineteenth century—to an epoch that comfortably predates modern professional science.^81^ At that time a single institution, under some strain, could still hold together the mechanisms of communication, approbation and networking that dominated British natural science. The story we have told here is partly that of just how long it was plausibly possible for the Royal Society to sustain this role in the rapidly changing scientific environment of the nineteenth century; but in the twenty-first, it is markedly less clear where such scientific leadership might plausibly come from.

## Notes

1 Ingen-Housz to Banks, 6 May 1782, in N. Chambers, *The scientific correspondence of Sir Joseph Banks, 1765–1820* (Pickering & Chatto, London, 2007) [hereafter *Banks scientific correspondence*], vol. 1, pp. 321–322.

2 Forbes to J. H. Symonds, January 1857, in J. C. Shairp, P. G. Tait and A. Adams-Reilly, *Life and letters of James David Forbes, FRS* (Macmillan, London, 1873), pp. 368–369.

3 See for example A. Goldgar, *Impolite learning: conduct and community in the Republic of Letters* (Yale University Press, New Haven, CT, 1995); S. Shapin and S. Schaffer, *Leviathan and the air-pump: Hobbes, Boyle and the experimental life* (Princeton University Press, Princeton, NJ, 1985); B. Bensaude-Vincent and C. Blondel (eds), *Science and spectacle in the European Enlightenment* (Ashgate, Aldershot, 2008); S. J. M. M. Alberti, ‘Conversaziones and the experience of science in Victorian England’, *Journal of Victorian Culture* **8**, 208–230 (2003).

4 A. J. Meadows, *The scientific journal* (Aslib, London, 1979); A. Fyfe, ‘Journals and periodicals’, in *A Companion to the History of Science* (ed. B. Lightman), pp. 387–399 (Wiley, New York, 2016).

5 M. Biagioli, ‘From ciphers to confidentiality: Secrecy, openness and priority in science’, *Brit. J. Hist. Sci.* **45** (2), 213–233 (2012), at 221.

6 For an overview of the history of the *Transactions*, see A. Fyfe, J. McDougall-Waters and N. Moxham, ‘350 years of scientific periodicals’, *Notes Rec.* **69**, 227–239 (2015).

7 Approved 18 February 1892, RS Council Minutes Printed [hereafter RS CMP] vol. 6 (first discussed in December 1891). The reading of papers merely in part or abstract was long-standing, as we will see, but 1892 appears to be the first acknowledgement of this practice and that the Secretaries could ‘read the title only’.

8 Jim Mussell makes a related point about the Chemical Society, as part of his analysis of the periodicity of science in the 1890s. See J. Mussell, *Science, time and space in the late nineteenth-century periodical press* (Ashgate, Aldershot, 2007), ch. 5.

9 See RS Statutes of 1663, ch. IV, reprinted in *The Record of the Royal Society of London for the improvement of natural knowledge* (Royal Society, London, 1940; repr. 1992), p. 289.

10 Shapin and Schaffer, *op. cit.* (note 3); S. Shapin, ‘The house of experiment in seventeenth-century England’, *Isis* **79**, 373–404 (1988); M. B. Hall, *Promoting experimental learning: experiment and the Royal Society, 1660–1727* (Cambridge University Press, 2002).

11 N. Moxham, ‘Fit for print: developing an institutional model of scientific periodical publishing in England, 1665–ca. 1714’, *Notes Rec.* **69**, 241–260 (2015).

12 RS Council Minutes (Originals) [hereafter RS CMO], vol. 4, pp. 48–55, 25 February 1752.

13 See I. Watts, ‘“We want no authors”: William Nicholson and the contested role of the scientific journal in Britain, 1797–1813’, *British Journal for the History of Science* **47**, 397–419 (2014).

14 RS CMO/8, 15 July 1802; on the uses of separate copies see also P. Corsi, ‘What do you mean by a periodical? forms and functions’, this issue.

15 W. H. Brock, ‘The development of commercial science journals in Victorian Britain’, in *The development of science publishing in Europe* (ed. A. J. Meadows), pp. 95–122 (Elsevier, Amsterdam, 1980); J. R. Topham, ‘Anthologizing the book of nature: the circulation of knowledge and the origins of the scientific journal in late Georgian Britain’, in *The circulation of knowledge between Britain, India, and China* (ed. B. Lightman, G. McOuat and L. Stewart), pp. 119–152 (Brill, Boston, 2013); G. Cantor, G. Dawson, G. Gooday, R. Noakes, S. Shuttleworth and J. R. Topham, *Science in the nineteenth-century periodical* (Cambridge University Press, 2004).

16 The issues of the new periodical were titled *Proceedings of the Royal Society*, but the title pages of early bound volumes say *Abstracts of the papers printed in the Philosophical Transactions* for continuity with the retrospective series of abstracts covering 1800–1830. The first issue included the meetings of 18 November to 16 December 1830, though its entry on the Royal Society's account does not appear in the printer's records until 25 February 1831: Taylor and Francis Journal (St Bride's Library) 1830–1840. The Council decision is recorded in RS CMO/12, p. 144, 16 December 1830.

17 RS CMP/6, 18 February 1892, clause 4.

18 John Hill, *A dissertation upon Royal Societies* (for John Doughty, London, 1750), *passim*, but especially pp. 16–31; George S. Rousseau, *The notorious Sir John Hill: the man destroyed by ambition in the age of celebrity* (Lehigh University Press, Bethlehem, PA, 2012), at pp. 65–85.

19 Still the best discussion of the Banks presidency of the Society is D. P. Miller, ‘The Royal Society of London 1800–1835: a case study in the cultural politics of scientific organization’, PhD Thesis, University of Pennsylvania (1981).

20 RS CMO/7, pp. 43, 45, 25 May and 15 June 1780.

21 A. Geikie, *Annals of the Royal Society Club: the record of a London dining-club in the eighteenth and nineteenth centuries* (Macmillan, London, 1917).

22 See for example Charles Blagden (Secretary to the Royal Society 1784–1797) to Banks, 23 February 1792, in *Banks scientific correspondence*, *op. cit*. (note 1), vol. 4, p. 108. For a discussion of the partial reading of mathematical papers in the context of the Banks presidency, see Benjamin Wardhaugh, ‘Charles Hutton and the “Dissensions” of 1783–1784: Scientific networking and its failures’, *Notes Rec.* **70** (2016). Published ahead of print: DOI: 10.1098/rsnr.2016.0016.

23 See M. B. Hall, *All scientists now: the Royal Society in the nineteenth century* (Cambridge University Press, 1984), pp. 2–4; C. Jungnickel and R. McCormmach, *Cavendish: the experimental life* (Bucknell University Press, Lewisburg, PA, 1999), pp. 335–351.

24 C. Creighton, ‘Ingen-Housz, Jan (1730–1799)’, rev. P. Fara, *Oxford dictionary of national biography* online ed. (Oxford University Press, 2004) http://www.oxforddnb.com/view/article/14388 (requires subscription; accessed 25 February 2016).

25 J. Priestley, *Experiments and observations relating to various branches of natural philosophy, with a continuation of the experiments upon air. The second volume* (printed by Pearson and Rollason for J. Johnson, London, Birmingham, 1781), pp. 180–191; R. E. Schofield, *The enlightened Joseph Priestley: a study of his life and work from 1773 to 1804* (Penn State University Press, University Park, PA, 2004), pp. 154–156; N. Beale and E. Beale, *Echoes of Ingen Housz: the long lost story of the genius who rescued the Habsburgs from smallpox and became the father of photosynthesis* (The Hobnob Press, Salisbury, 2011), pp. 310–318. On the contested nature and European context of early eudiometric experiments, see S. Schaffer, ‘Measuring virtue: eudiometry, enlightenment and pneumatic medicine’, in *The medical enlightenment of the eighteenth century* (ed. A. Cunningham and R. French), pp. 281–318 (Cambridge University Press, 1990).

26 *Critical Review* **52**, 133–141 and 176–184 (July 1781).

27 Ingen-Housz to Banks, 6 May 1782, in *Banks scientific correspondence*, *op. cit*. (note 1), vol. 1, pp. 321–322.

28 Banks to Ingen-Housz, 25 May 1782, *Banks scientific correspondence*, *op. cit*. (note 1) vol. 1, pp. 326–327.

29 Banks to Blagden, 16 June 1782, *Banks scientific correspondence*, *op. cit*. (note 1) vol. 1, p. 330.

30 Blagden to Banks, 30 June 1782, *Banks scientific correspondence*, *op. cit*. (note 1) vol. 1, p. 333.

31 Jan Ingen-Housz, ‘Some farther considerations on the influence of the vegetable kingdom on the animal creation. By John Ingen-Housz, Counsellor to the Court, and Body Physician to the Emperor, F. R. S. &c.’, *Phil. Trans. R. Soc. Lond.* **72**, 426–439 (1782). The criticisms of Priestley are mostly confined to the first six pages of the manuscript, which are struck out, probably reflecting Banks's cuts; a manuscript note to the printer instructs him to begin the composition at page six. RS Letters & Papers 70/259.

32 RS Blagden Papers CB/3/3 f.77v (17 November 1795), for instance.

33 John Tyndall was a member of both the RS Philosophical Club and the Metropolitan Red Lions; and Tyndall's correspondence also mentions RS soirees and breakfasts, e.g. Tyndall to Hirst, n.d. [late May/early June 1852], Royal Institution (RI) MS JT/1/HTYP/195-196; Tyndall to Hirst, 9 Mar. 1856, RI MS JT/1/T/HTYP/441. All quotations from Tyndall's correspondence are taken from the Tyndall Correspondence Project's digital transcriptions. For less formal activities, including taverns, smoking and singing, see H. Gay and J. W. Gay, ‘Brothers in science: science and fraternal culture in nineteenth-century Britain’, *Hist. Sci.* **35**, 425–453 (1997).

34 T. G. Bonney, *Annals of the Philosophical Club of the Royal Society, written from its minute books* (Macmillan, London, 1919), esp. pp. vi and 1–2.

35 The motion to allow discussion was passed by Council on 5 June 1845, RS CMP/1, p. 501. A similar proposal had failed in 1836, see M. B. Hall, *op. cit.* (note 23), p. 69. The propensity for heated debate at the Geological Society is encapsulated in the title of J. C. Thackray (ed.), *To see the fellows fight: eye witness accounts of meetings of the Geological Society of London and its Club, 1822–1868*, reissue ed. (British Society for the History of Science, Oxford, 2003).

36 Tyndall to T. A. Hirst, 9 January 1853, RI MS JT/1/HTYP/224–226. This episode is discussed in R. Jackson, ‘John Tyndall and the early history of diamagnetism’, *Ann. Sci.* **72**(4), 435–489 (2015), at pp. 23–24.

37 Tyndall to T. A. Hirst, 9 January 1853, RI MS JT/1/HTYP/224–226.

38 By 1858 the *Literary Gazette*'s list of meetings for the coming week included paper titles and authors for Royal Society meetings. It had not done so in 1857.

39 Tyndall to Spottiswoode, 24 December 1881, RI MS JT/TYP/2/1286.

40 David Kronick analysed the relative growth of scientific periodicals in Germany, France and Britain in the eighteenth century, finding much more significant growth in Germany: see D. A. Kronick, *A history of scientific & technical periodicals: the origins and development of the scientific and technical press, 1665–1790* (Scarecrow Press, Metuchen, NJ, 1976), pp. 89–99.

41 For other accounts that place learned society meetings in their wider intellectual culture, see D. A. Finnegan, ‘The work of ice: glacial theory and scientific culture in early Victorian Edinburgh’, *Brit. J. Hist. Sci.* **37**, 29–52 (2004); Mussell, *op. cit.* (note 8), ch. 5.

42 An earlier controversy involving Forbes and glaciers has been discussed in C. Smith, ‘William Hopkins and the shaping of dynamical geology: 1830–1860’, *Brit. J. Hist. Sci.* **22**, 2-52 (1989), at pp. 43–51. See also the broader account that sets Forbes's works on glaciers in the context of Victorian alpinism and field science in B. Hevly, ‘The heroic science of glacier motion’, *Osiris* **11**, 66–86 (1996).

43 Huxley later claimed that his only contribution to the joint paper was that he suggested Tyndall should investigate glaciers, see L. Huxley (ed.), *Life and letters of Thomas Henry Huxley* (Macmillan, London, 1900), vol. 1, p. 144.

44 Tyndall to Hirst, 4 December 1856, RI MS JT/1/HTYP/484–484a.

45 For glacial theory, see J. S. Rowlinson, ‘The theory of glaciers’, *Notes Rec. R. Soc. Lond.* **26**, 189–204 (1971) and M. Rudwick, *Worlds before Adam: the reconstruction of geohistory in the age of reform* (University of Chicago Press, 2008), pp. 34–36. For the North British connection, see C. Smith, *The science of energy: a cultural history of energy physics in Victorian Britain* (Athlone Press, London, 1998).

46 Bonney, *op. cit.* (note 34), pp. 133–134.

47 J. Tyndall and T. H. Huxley, ‘On the structure and motion of glaciers’, *Phil. Trans. R. Soc. Lond.* **147**, 327–346 (1857).

48 See Register of Papers RS MS421, 1856/57, items 223–229.

49 Register of Papers, RS MS421, 1856/57, item 231.

50 Tyndall to Hirst, 16 January 1857, RI MS JT/1/T/636.

51 Darwin to Huxley, 17 January 1857, Darwin Correspondence Project, ‘Letter no. 2041’, http://www.darwinproject.ac.uk/DCP-LETT-2041 (accessed 25 February 2016).

52 On 7 March, Tyndall told Forbes that the paper was with the referees: see Tyndall to Forbes, 7 March 1857, University of St Andrews, J. D. Forbes Papers, msdep7, Incoming letters 1857, no. 33. That Tyndall knew the decision by late May is apparent from Tyndall to Clausius, 28 May 1857, RI MS JT/1/T/174. Intriguingly, the Royal Society's ‘Register of Papers’ does not give the names of any referees for this paper, which may indicate that it was examined by George Stokes (Secretary) alone. On Stokes as a referee of Tyndall's later papers, see M. Baldwin, ‘Tyndall and Stokes: correspondence, referee reports and the physical sciences in Victorian Britain’, in *The age of scientific naturalism: Tyndall and his contemporaries* (ed. B. Lightman and M. S. Reidy), pp. 171–186 (Pickering & Chatto, London, 2014).

53 The parts usually appeared around May and November; but the printers’ bill for 1857 part II was not issued until February 1858: Taylor & Francis Journals, 1857–1861, 18 February 1858, St Bride's Library.

54 Forbes to J. H. Symonds, [?] January 1857, in Shairp, Tait and Adams-Reilly, *op. cit.* (note 2), p. 369. Shairp gives the date as 3 January, but, as Rowlinson argues, it must be after 15 January, see Rowlinson, *op. cit.* (note 45), p. 193 and n39.

55 Forbes to J. H. Symonds, January 1857, in Shairp, Tait and Adams-Reilly, *op. cit.* (note 2), p. 369.

56 The RI lecture was advertised in *Athenaeum* (17 January 1857), p. 86. It was reported in *Saturday Review* (31 January 1857), pp. 102–103, and in *Literary Gazette* (7 February 1857), 135–136. Only the latter mentioned the RS paper, in addition to the RI lecture.

57 ‘Royal Society’, *Philosophical Magazine* (September 1857), p. 220.

58 Tyndall to Clausius, 9 February 1857, RI MS JT/1/T/172.

59 Matteucci to Tyndall, 19 February 1857, RI MS JT/1/M/62.

60 Tyndall to de la Rive, 13 March 1857, RI MS JT/1/TYP/1/1/347.

61 Tyndall to Herschel, 2 March 1857, RI MS JT/1/TYP/2/503. Herschel responded with interest, seeking clarification and suggesting a further experiment, see Herschel to Tyndall, 17 March 1857, RS Herschel Papers, HS/17/386.

62 See ‘The month: science and arts’, *Chambers's Journal* (28 February 1857), p. 141. This column was usually supplied by Walter White, librarian of the Royal Society. See A. McConnell, ‘White, Walter (1811–1893)’, *Oxford dictionary of national biography* online ed. (Oxford University Press, 2004) http://www.oxforddnb.com/view/article/29277 (requires subscription; accessed 27 February 2016).

63 It is difficult to date when *Proceedings* was actually issued. Taylor & Francis printed two issues on 5 and 23 February. Unless Forbes was slow to react, the glacier paper was presumably in the second of these issues.

64 Forbes to Tyndall, 6 March 1857, RI MS JT/1/TYP/1/3941.

65 Tyndall to Forbes, 7 March 1857, in University of St Andrews, J. D. Forbes Papers, msdep7, Incoming letters 1857, no. 33.

66 Reported in ‘Prof. Forbes on some properties of ice near its melting point’, *Athenaeum* (21 August 1858) and in *Proc. R. Soc. Edinb.* **4**, 103–106 (1857–1862) (19 April 1858).

67 Forbes thanked Tyndall for his copy of the paper on 23 October, Forbes to Tyndall, 23 October 1857, RI MS JT/1/TYP/12/3942. On slowness, see Tyndall to Clausius, 28 May 1857, RI MS JT/1/T/174.

68 Titles and authors (only) were reported in *Athenaeum* (21 February 1857), p. 249.

69 On the development of peer review practices, see D. A. Kronick, ‘Peer review in 18th-century scientific journalism’, *J. Am. Med. Assn* **263**, 1321 (1990); and M. Biagioli, ‘From book censorship to academic peer review’, *Emergences: Journal for the Study of Media & Composite Cultures* **12** (1), 11–45 (2002). Our own study of the Royal Society's editorial practices is currently under review.

70 Edward Sabine to Tyndall, 16 January [1869], RI MS JT/1/TYP/4/1324.

71 M. B. Hall, *op. cit.* (note 23), p. 2. The dissenting and reformist cliques of 1783 and the 1820s and 1830s were led by mathematicians in particular (though see Wardhaugh, *op. cit.* note 22, for a more nuanced discussion of what was actually at stake in the 1783 ‘Dissensions’).

72 On the faster speed of scientific news and publication by the late nineteenth century, see Mussell, *op. cit.* (note 8) and M. Baldwin, ‘“Keeping in the race”: physics, publication speed and national publishing strategies in *Nature*, 1895–1939’, *Brit. J. Hist. Sci.* **47**, 257–279 (2014).

73 The paper was by J. E. Gray on fossil chitons. See Council Minutes for 25 May 1848, RS CMP/2, 78–79; and 6 July 1848, RS CMP/2, 80–87 (when it was discovered that the changes were, in fact, ‘merely verbal’).

74 RS CMP/5, 13 May 1880.

75 M. Baldwin, *Making* Nature*: the history of a scientific journal* (University of Chicago Press, 2015), pp. 65–66.

76 J. Morrell and A. Thackray, *Gentlemen of science: early years of the British Association for the Advancement of Science* (Oxford University Press, 1981).

77 Fyfe, McDougall-Waters and Moxham, *op. cit.* (note 6).

78 R. Porter, ‘Gentlemen and geology: the emergence of a scientific career, 1860–1920’, *Hist. J.* **21**, 809–836 (1978), at p. 815; J. A. Secord, ‘How scientific conversation became shop talk’, in *Science in the marketplace: nineteenth-century sites and experiences* (ed. A. Fyfe and B. Lightman), pp. 23–59 (University of Chicago Press, Chicago, IL, 2007).

79 On the late adoption of peer review by *Nature*, see M. Baldwin, ‘Credibility, peer review, and *Nature*, 1945–1990’, *Notes Rec.* **69**, 337–352 (2015).

80 The propensity to use a count of publications as a marker of reputation might be dated to Charles Babbage's *Decline of science in England* (1830), but it was not then normal practice. On counting publications, see A. Csiszar, ‘Broken pieces of fact: the scientific periodical and the politics of search in nineteenth-century France and Britain’ (unpublished PhD dissertation, Harvard, 2010), ch. 3.

81 For the extensive free circulation of knowledge enabled by the *Philosophical Transactions* in the eighteenth and nineteenth centuries, see A. Fyfe, ‘Journals, learned societies and money: *Philosophical Transactions*, *ca.* 1750–1900’, *Notes Rec.* **69**, 277–299 (2015).

